# Supplementary material for: How Rh surface breaks CO2 molecules under ambient pressure
Source: Nat Commun. 2020 Nov 6;11:5649. doi: 10.1038/s41467-020-19398-1 (PMC7648795; doi:10.1038/s41467-020-19398-1)
Supplement: Supplementary file 1 — Supplementary Information [file 41467_2020_19398_MOESM1_ESM.pdf]

Supplementary Information

How Rh surface breaks CO_2 molecules under ambient pressure

Jeongjin Kim[1,8], Hyunwoo Ha[2,8], Won Hui Doh[1,8], Kohei Ueda[3], Kazuhiko Mase[4], Hiroshi Kondoh[3], Bongjin Simon Mun[5,6]*, Hyun You Kim[2]* and Jeong Young Park[1,7]*

[1]Center for Nanomaterials and Chemical Reactions, Institute for Basic Science (IBS), Daejeon 34141, Republic of Korea.

[2]Department of Materials Science and Engineering, Chungnam National University, Daejeon 34134, Republic of Korea.

[3]Department of Chemistry, Keio University, 3-14-1 Hiyoshi, Kohoku-ku, Yokohama 223-8522, Japan.

[4]Institute of Materials Structure Science, High Energy Accelerator Research Organization, SOKENDAI (The Graduate University for Advanced Studies), 1-1 Oho, Tsukuba 305-0801, Japan.

[5]Department of Physics and Photon Science, School of Physics and Chemistry, Gwangju Institute of Science and Technology (GIST), Gwangju 61005, Republic of Korea.

[6]Center for Advanced X-ray Science, GIST, Gwangju 61005, Republic of Korea.

[7]Department of Chemistry, Korea Advanced Institute of Science and Technology (KAIST), Daejeon 34141, Republic of Korea.

[8]These authors contributed equally: Jeongjin Kim, Hyunwoo Ha, Won Hui Doh.

*Corresponding author e-mail: bsmun@gist.ac.kr; kimhy@cnu.ac.kr; jeongypark@kaist.ac.kr.

The supplementary information includes:

Supplementary Fig. 1-17

Supplementary Table 1-4

Supplementary References

Table of Contents

Supplementary Figures

1: NAP-STM observation of (2 × 2) structure formation under 0.1 mbar CO environment…….…..…3

2: NAP-STM observations of l-CO_2 and b-CO_2 molecules under 0.1 mbar CO_2 environment……….4

3: NAP-STM observations of physisorption and chemisorption events of CO_2 molecules……………5

4: Representative line profile analysis results of CO_2(ads.) at different tunneling conditions………..6

5: Simulated STM image of adsorbate b-CO_2 molecule on the Rh(111) surface………………………7

6: LEED pattern analysis and atomistic ball model of clean Rh(111) surface…………………………8

7: Comparison plots of Rd 3*d* core-level NAP-XP spectra under UHV, CO(g), and CO_2(g)……….…9

8: Adsorbate-sensitive C 1*s* core-level NAP-XP spectrum at 0.1 mbar CO_2…………………………11

9: NAP-XPS analysis of O 1*s* core-level at each different sample measurement position…………...12

10: Comparison of initial and equilibrium O 1*s* core-level spectra at 0.1 mbar CO_2……………….....13

11: Acquired C 1*s* core-level spectra of before and after pump down…………………………….….14

12: Complex adsorbate structures of CO_2(ads.) and its intermediates under 0.1 mbar CO_2…………15

13: Unnormalized C 1*s* core-level spectra of Figure 3d and histogram analysis……………………..16

14: NAP-STM observation on the Rh(111) surface in 1 × 10^{-6} mbar CO_2 condition at 300 K………17

15: Constructed model structures of before and after CO_2 dissociation on Rh surface……………....20

16: DFT-calculated adsorption energy change between one and four CO_2 molecules…...…………..21

17: A constructed 6×6×4 supercell of the slab model of Rh in DFT calculations…………………..……22

Supplementary Table

1: The comparison of deconvoluted peaks signal intensity ratios in NAP-XPS measurements……..10

2: Detailed peak deconvolution profiles of CO_2(ads.) species in O 1*s* core-level NAP-XP spectra….12

3: Previously reported activation energy barriers of the CO_2 dissociation process over Rh surface.....18

4: Calculated lattice parameters of Rh by using various exchange-correlation functionals………....19

Supplementary References……….……………………………………………………….………..23

0.1 mbar CO environment (T = 300 K)

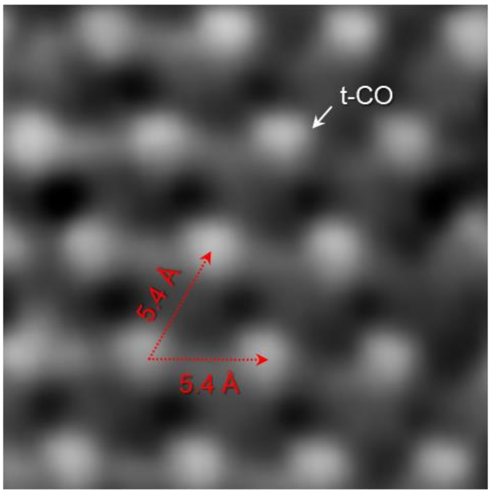

V_t = 0.78 V; I_t = 0.16 nA

Supplementary Figure 1. Atom-resolved adsorbate structure characterization under 0.1 mbar CO environment using NAP-STM. The observed pattern of (2 × 2) structures at the corner of bright protrusions has a nearest-neighborhood distance of 5.4 Å. Adsorbate CO molecules occupy both atop and hollow sites of Rh(111) surface together at 300 K, at which protrusion contrasts would be resolved depending on the tunneling electrons measurement conditions.

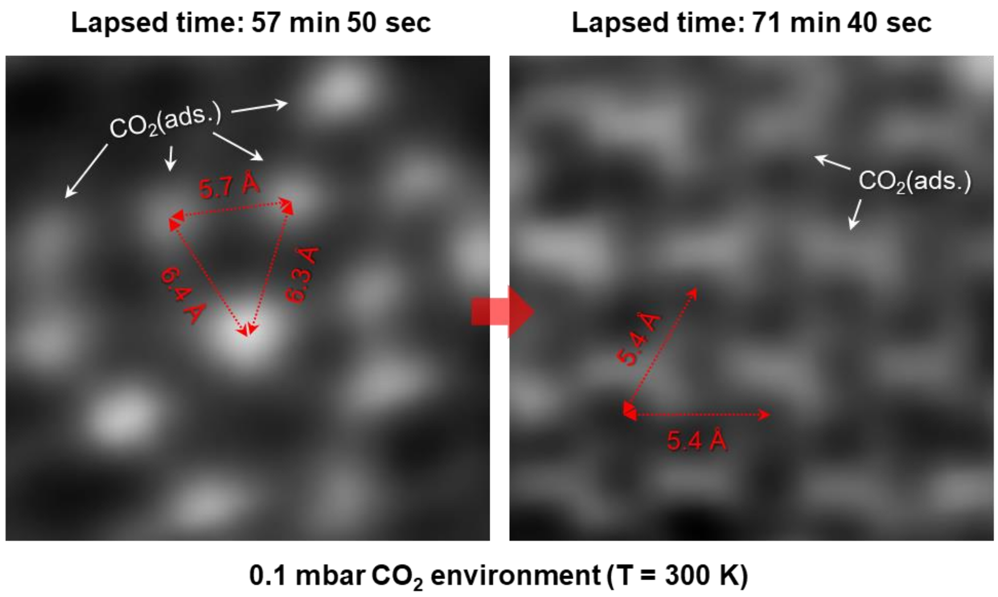

Supplementary Figure 2. NAP-STM measurement results of adsorbate l-CO_2 (left) and b-CO_2 (right) molecules on the Rh(111) surface under 0.1 mbar CO_2 environment at 300 K. The physisorption process of l-CO_2 molecules shows a trend of quickly diffusing motion at a given rate on the surface, which leads to randomly arranged weakly bound molecular adsorption, involving the different nearest-neighborhood distances, as indicated by arrows (red color). In contrast, the chemisorption process involved the partially ordered arrangements of b-CO_2 molecules at the same place. The partially ordered structure lies on the surface with the periodic distance of 5.4 Å on the Rh(111) surface.

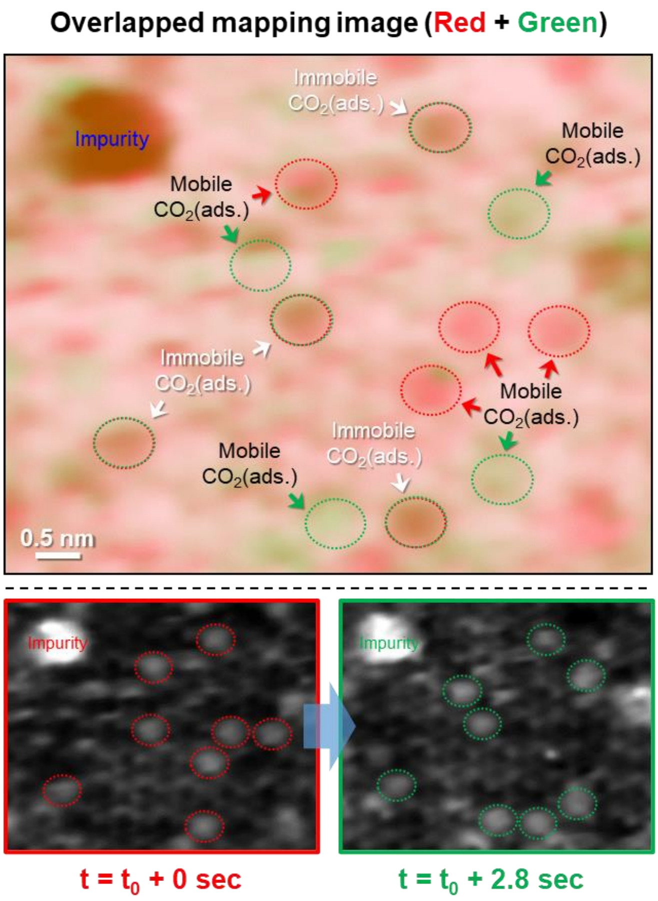

Supplementary Figure 3. Atomic-scale observations of physisorption and chemisorption CO_2 molecules on the Rh(111) surface at 0.1 mbar CO_2. The representative NAP-STM image enclosed by a rectangle of red or green color (acquisition time interval; Δt = 2.8 sec) demonstrates that different features of mobile and immobile bright blobs simultaneously in the overlapped mapping image, which proves characterized distinctive features of CO_2(ads.) from the impurity at the same local area.

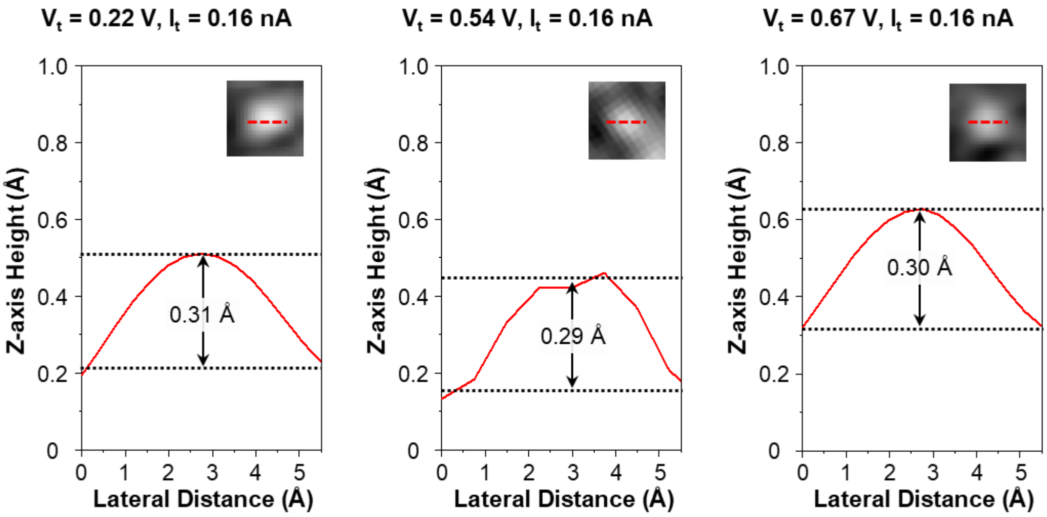

Supplementary Figure 4. Representative line profile analysis results of the characterized CO_2(ads.) molecule at different tunneling conditions of 0.22, 0.54, and 0.67 V at 0.1 mbar CO_2 environment, which indicates that the recorded NAP-STM images of CO_2(ads.) molecules are irrelevant to the STM tip-induced artifact between 0.22 and 0.67 V of positively biased voltage.

DFT-simulated STM image

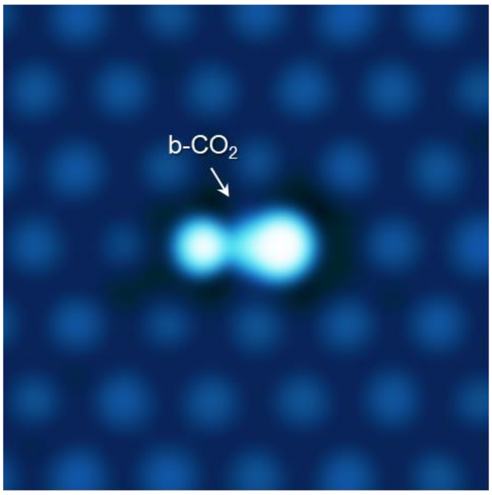

b-CO_2 chemisorption on Rh(111)

Supplementary Figure 5. A top view of the DFT-simulated STM image demonstrates that the linear geometry of chemisorbed b-CO_2 molecule occupies atop and bridge sites of Rh surface each for partial bonding of O−CO* in the same plane.

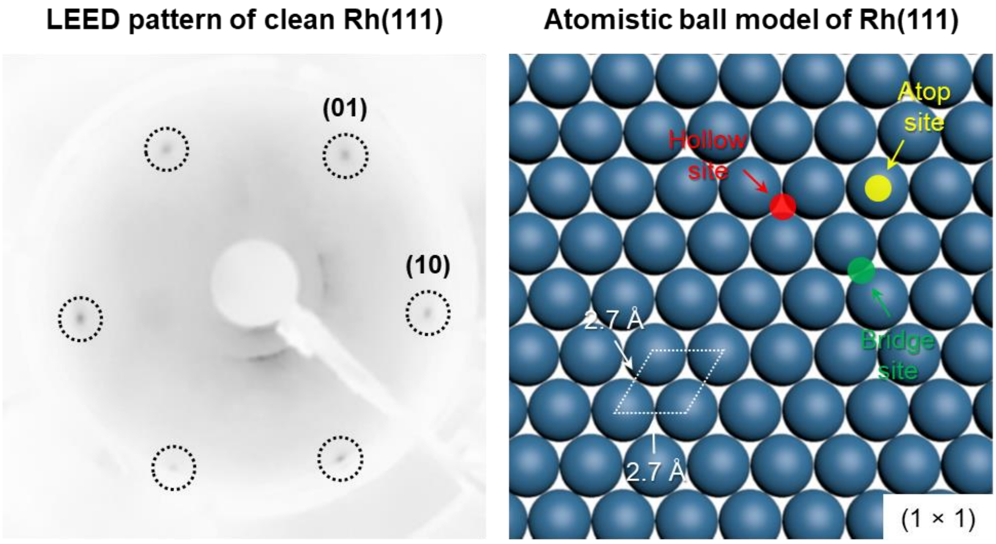

Supplementary Figure 6. Acquired LEED pattern of clean Rh(111) surface in UHV (left) and atomistic ball model of Rh(111) surface (right). The resolved spots of LEED pattern make a hexagonal structure in reciprocal lattice space, which could be interpreted as a periodically arrayed (1 × 1) structure in real space. The theoretical model of Rh(111) surface visualizes that the ordered structure has the nearest-neighborhood distance of 2.7 Å between Rh atoms.

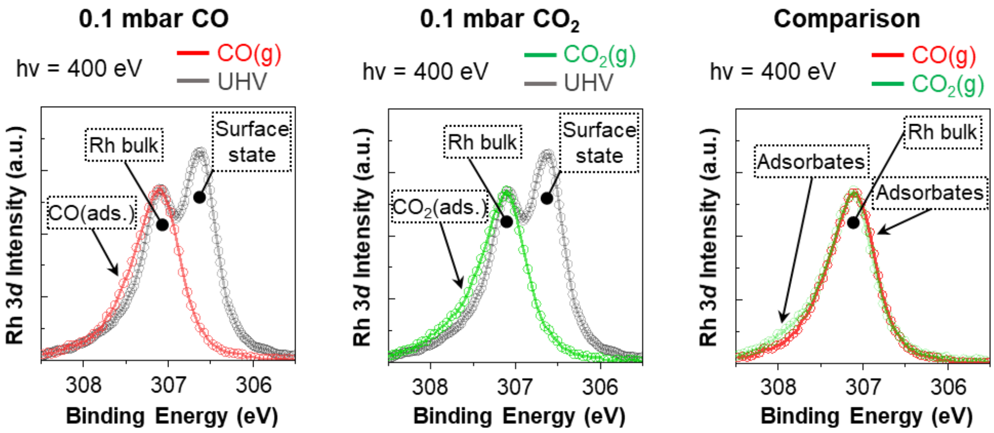

Supplementary Figure 7. The collected comparison plots of Rh 3*d* core-level NAP-XP spectra under UHV, 0.1 mbar CO, and 0.1 mbar CO_2. Each acquired spectrum in gaseous condition is compared with the measurement result on the clean Rh(111) surface in UHV (left and center), and then, the Rh 3*d* core-level spectra in gas environments are also plotted as the overlapping comparison (right) to show a delicate spectral difference depending on the kinds of gas molecules collisions at NAP.

**NAP-XPS Gas environment**	**Chemical Equilibrium**	**C 1*s* Peak Intensity ratio**	**O 1*s* Peak Intensity ratio**
0.1 mbar CO	Equilibrium	0.51 (t-CO/h-CO)	0.80 (t-CO/h-CO)
0.1 mbar CO_2	Initial	0.70 (C2/C1)	1.05 (O3/O2)
	Equilibrium	1.21 (C2/C1)	1.68 (O3/O2)

Supplementary Table 1. Comparison of relative NAP-XPS signal intensities ratio of deconvoluted peaks obtained in 0.1 mbar CO and CO_2 conditions.

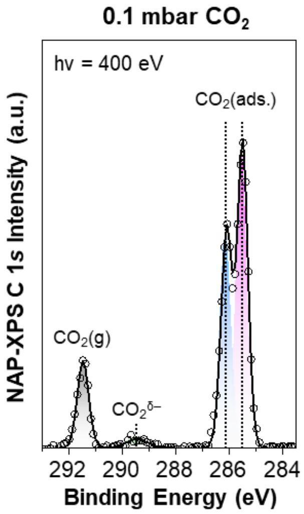

Supplementary Figure 8. Adsorbate-sensitive C 1*s* NAP-XP spectrum under 0.1 mbar CO_2(g) environment at a different distance of sample-to-aperture. This spectroscopic result was measured after the gas exposure of 8 min at 300 K. The assigned peaks of CO_2(ads.), $CO_2^{\delta-}$, and CO_2(g) are corresponding to chemisorption, physisorption, and scattered gas molecule species. Contrary to Figure 2b, the CO_2(ads.) species for chemisorption is also assigned at 286.1 eV instead of the CO(dis.), because the CO_2 dissociation process is not yet fully triggered at that time.

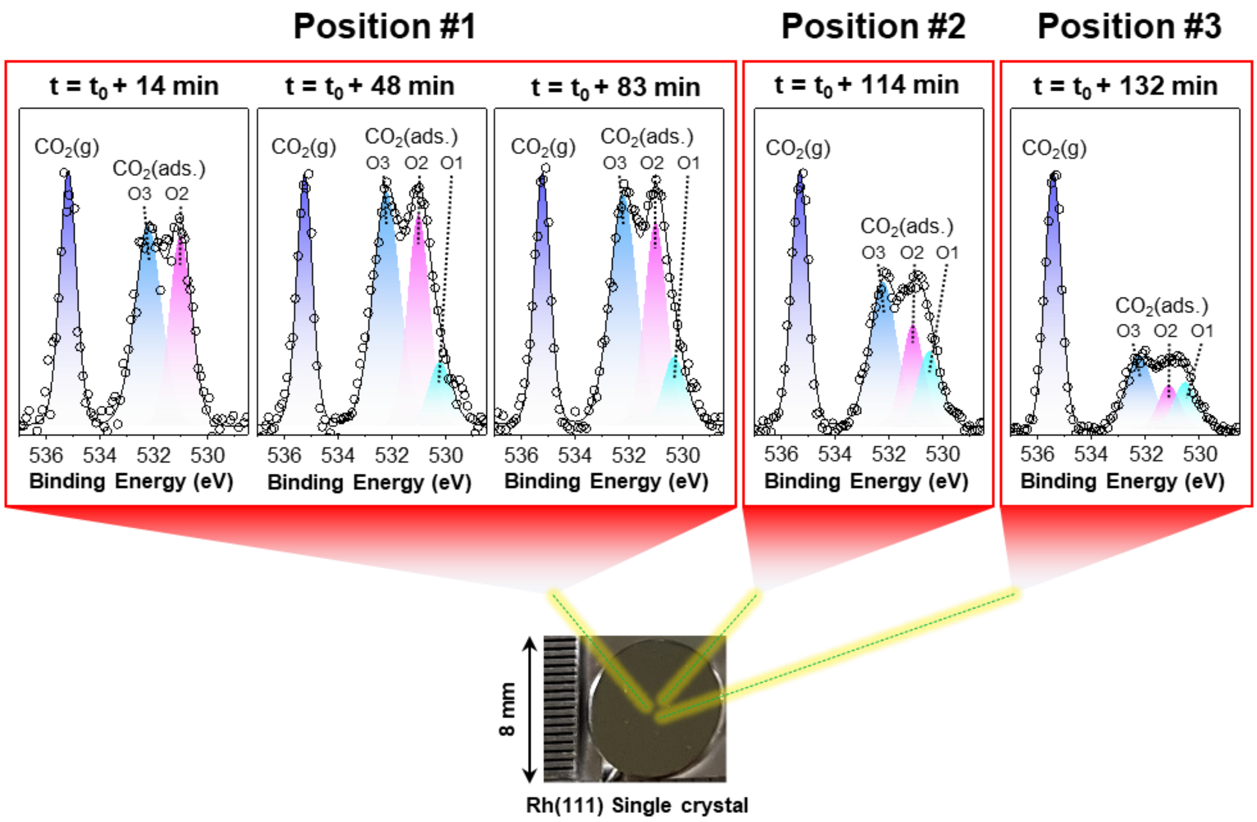

Supplementary Figure 9. Sequentially acquired O 1*s* core-level NAP-XPS measurement results under 0.1 mbar CO_2 environments at 300 K. At sample position #1, we could observe two of b-CO_2(ads.) peaks at early stage of overall measurements ($t = t_0 + 14$ min); then, a new peak appeared as labeled O1 with other peaks (O2 and O3). At different sample measurement positions, such as #2 and #3, we collected a similar trend of peak deconvolution profiles in O 1*s* core-level spectra under the CO_2 environment. It means that the photoionization process or photon-induced surface damaged artifacts can be excluded from our NAP-XPS analysis results.

Lapsed time (min)	Peak Center Position (eV)			FWHM (eV)			Integrated Area (%)		
	O1	O2	O3	O1	O2	O3	O1	O2	O3
14	N/A	531.0	532.2	N/A	0.96	1.20	N/A	43.3	56.7
48	530.2	531.0	532.2	1	0.96	1.20	11.7	37	51.3
83	530.3	531.0	532.2	1	0.96	1.20	12.7	36.3	51.1
114	530.5	531.1	532.2	1	0.96	1.20	21.7	28.3	50
132	530.5	531.1	532.2	1	0.96	1.20	26.3	23.8	49.9

Supplementary Table 2. Detailed peak deconvolution profiles of O1, O2, and O3 in O 1*s* core-level spectra during time-lapse NAP-XPS measurements under 0.1 mbar CO_2 environment at 300 K.

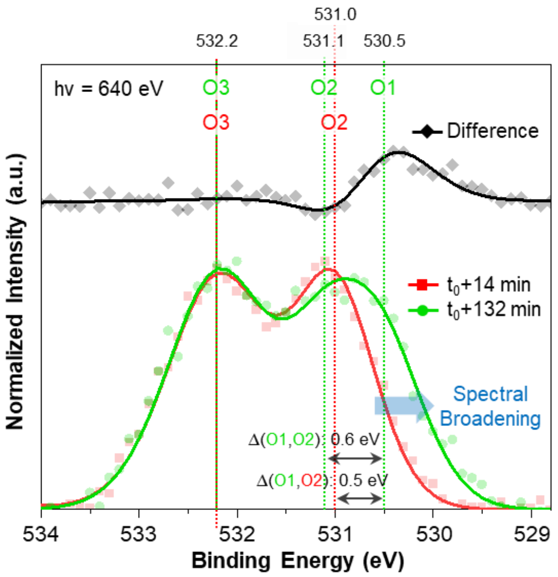

Supplementary Figure 10. The comparison of initial (t_0 + 14 min) and equilibrium (t_0 + 132 min) O 1*s* core-level spectra under 0.1 mbar CO_2 environment. The plotted spectra show a remarkable difference of signal intensity at around 530.5 eV, because there is a significant spectral broadening feature. The measured peak-to-peak difference of binding energy between O1 and O2; i.e. Δ(O1,O2), is 0.5–0.6 eV, which provides evidence of intermediate species (O1) evolution by CO_2 dissociation process at equilibrium.

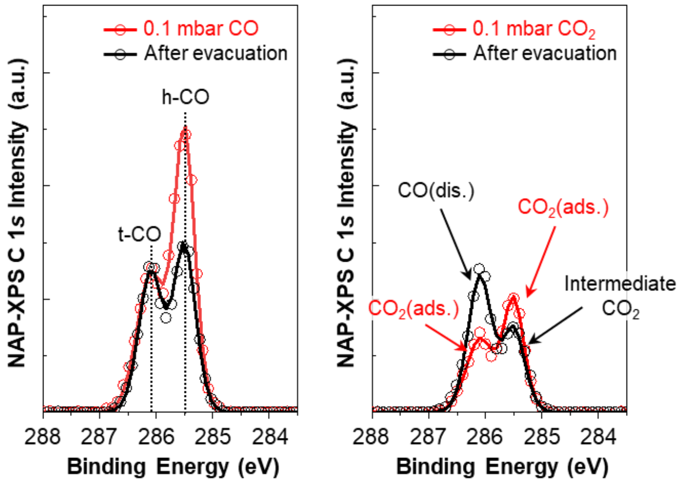

Supplementary Figure 11. Acquired C 1*s* core-level NAP-XPS measurements (hν = 400 eV) at the Rh(111) interface under 0.1 mbar CO and CO_2 (red color), and after pump down (black color). The comparably plotted reactant gas-in and gas-out spectra in each different environment demonstrate the change of adsorbate coverage on adsorption sites consisting of Rh atoms. Representative photo-induced contaminants, such as atomic carbon and carbidic species, did not appear during the NAP-XPS analysis.

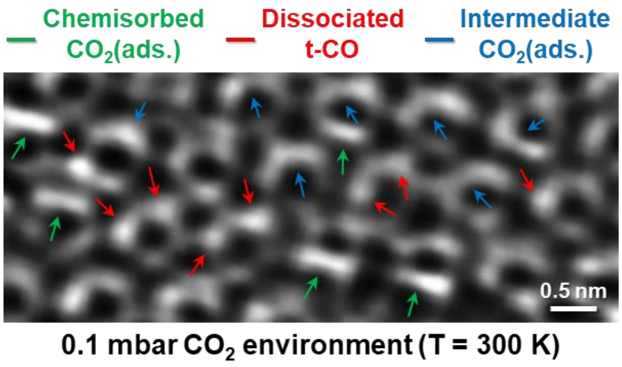

Supplementary Figure 12. The complex structures consisting of chemisorbed CO_2(ads.), dissociated t-CO, and intermediate CO_2(ads.) during the catalytic CO_2 dissociation on the Rh(111) surface. Each species indicated by the arrow with a corresponding color of green, red, or blue shows the randomly formed atomic structures, because the catalytic reaction process could make the complex interactions between adjacent adsorbate CO_2 molecules and surface Rh atoms in transition state.

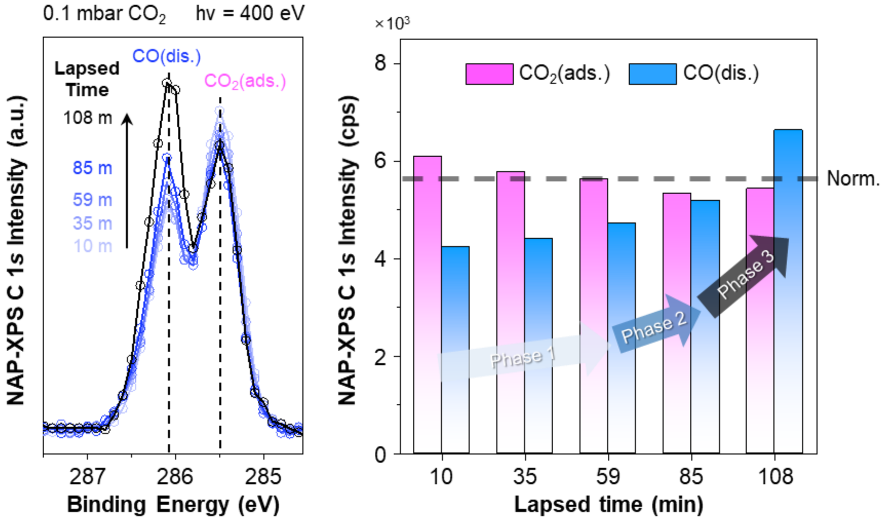

Supplementary Figure 13. The recorded peak intensity changes of CO(dis.) and CO_2(ads.) in C 1*s* core-level NAP-XPS measurements and its histogram analysis results. The unnormalized spectra show the gradual increment of dissociated CO from CO_2 over Rh(111) surface, but the measured peak intensities of CO_2(ads.) in time-lapse signal collections are unrelated to the trend of CO(dis.) as a function of lapsed time. Excess amounts of CO_2(g) were backfilling in the analysis chamber when the C 1*s* core-level spectrum was acquired at each shot of X-ray photon beam irradiation.

1 × 10^{-6} mbar CO_2 environment (T = 300 K)

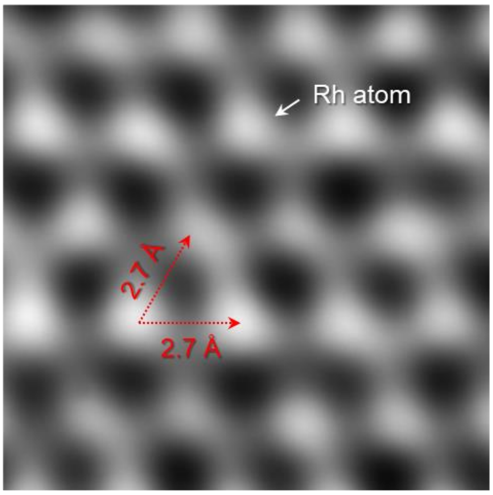

V_t = 0.21 V; I_t = 0.30 nA

Supplementary Figure 14. A direct observation image of hexagonal Rh atom arrays in atom-resolved resolution under 1 × 10^{-6} mbar CO_2 environment at 300 K. The bright spot indicated by an arrow (white color) has an identical nearest-neighborhood distance of 2.7 Å with the cleaned Rh(111) surface. Even though the exposed CO_2(g) molecules constantly collide with Rh atoms in the reaction cell, we could not observe significant topographic changes in the low pressure of CO_2 condition because of the extremely low sticking probability.

	**This work (theory)**	Abbott et al.[1] (theory)	Liu et al.[2] (theory)	Ko et al.[3] (theory)	Goodman et al.[4] (experiment)
Method	**RPBE+D3**	PC-MURT	PBE	PBE+D2	H_2/CO_2 mixture $p(CO_2)$ = 1 Torr
ΔE_a (eV)	**0.58**	0.79	0.50	0.56	0.74

Supplementary Table 3. Previously reported activation energy barriers (ΔE_a) of the CO_2 dissociation over Rh surfaces by theoretical calculation and experiment.

	Experiment	HSE06	PBE	PW91	**RPBE (This work)**
Lattice parameter (Å)	3.80	3.79	3.83	3.85	**3.85**

Supplementary Table 4. Lattice parameters of Rh calculated by various exchange-correlation functionals. The RPBE exchange-correlation functional is known to be overestimating the lattice parameter of late-transition metals. The calculated lattice parameter of Rh (3.85 Å) was overestimated by 0.05 Å from the experimental value. The higher-level exchange-correlation treatment methods such as the random phase approximation (RPA)[5] or the RPA + exact exchange scheme[6] have provided the more accurate surface properties. On the other hand, among the conventional GGA-level exchange-correlation functionals, the use of RPBE gives relatively reasonable prediction results of the binding energy of CO on late-transition metals[7]. Because our main purpose was understanding the dissociation mechanism of CO_2 on Rh(111) and reproducing NAP-STM images of multiple CO binding configuration, we adopted the RPBE functional and combined the D3 vdW-correction method suggested by Klimeš and Michaelides[8] as a compromise between the calculation accuracy and the computational cost.

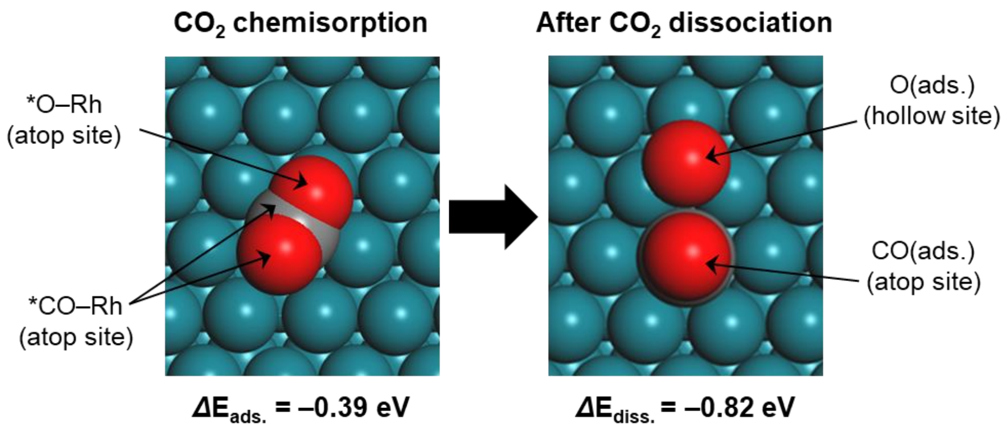

Supplementary Figure 15. DFT-calculated morphology of the chemisorbed b-CO_2 before and after dissociation on the Rh(111) surface (top view). The negative dissociation energy of –0.82 eV indicates that the chemically adsorbed b-CO_2 can be spontaneously dissociated into *CO−Rh (atop site) and *O−Rh (hollow site). The *O–Rh of the *b*-CO_2 initially bound at the bridge site was transferred to the hollow site upon dissociation.

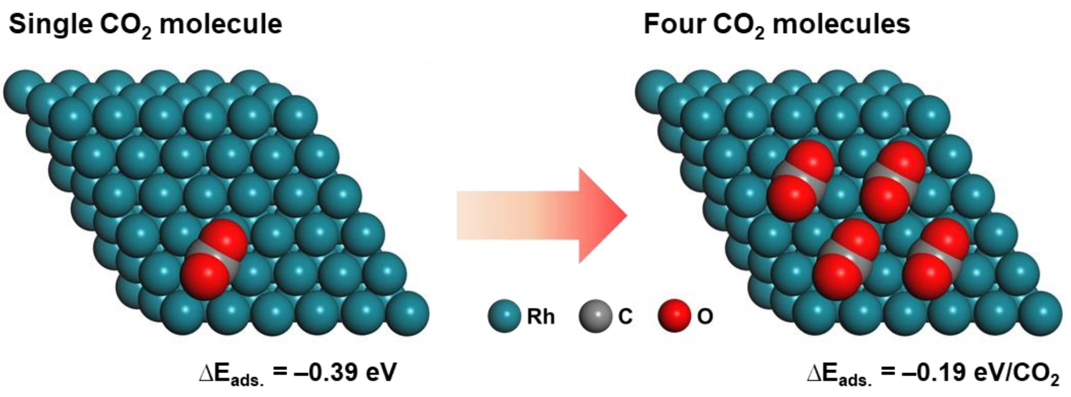

Supplementary Figure 16. The DFT-calculated adsorption energy change between one and four CO_2 molecules configurations on a {111} facet of Rh surface. Both calculated $\Delta E_{ads.}$ values represent the average binding energy per CO_2 molecule, respectively.

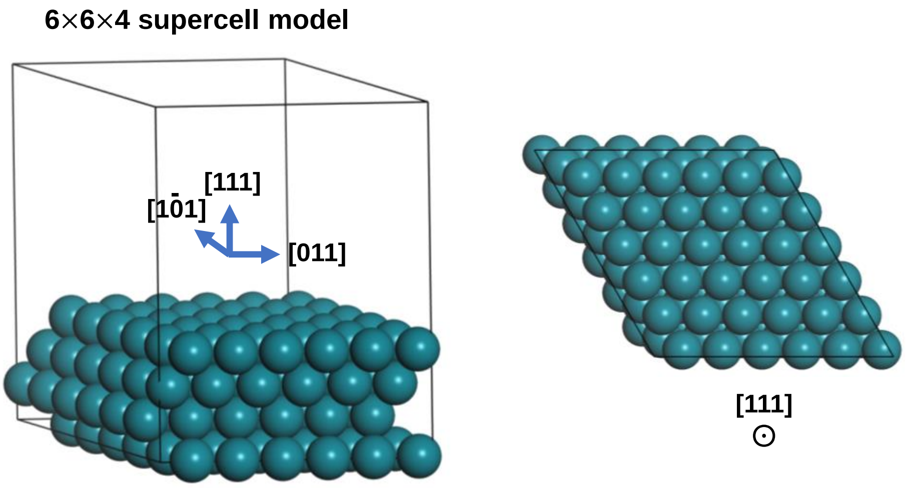

Supplementary Figure 17. A constructed 6×6×4 supercell of the slab model of Rh in DFT calculations. A perspective projection view of the Rh model structure (left) and its top view image along to a direction of [111] (right) show the three-dimensional configurations of the Rh slab model consisting of 144 atoms.

Supplementary References

1 Abbott, H. L. & Harrison, I. Activated Dissociation of CO_2 on Rh(111) and CO Oxidation Dynamics. *J. Phys. Chem. C* **111**, 13137-13148 (2007).

2 Liu, X., Sun, L. & Deng, W.-Q. Theoretical Investigation of CO_2 Adsorption and Dissociation on Low Index Surfaces of Transition Metals. *J. Phys. Chem. C* **122**, 8306-8314 (2018).

3 Ko, J., Kim, B.-K. & Han, J. W. Density Functional Theory Study for Catalytic Activation and Dissociation of CO_2 on Bimetallic Alloy Surfaces. *J. Phys. Chem. C* **120**, 3438-3447 (2016).

4 Goodman, D. W., Peebles, D. E. & White, J. M. CO_2 dissociation on rhodium: Measurement of the specific rates on Rh(111). *Surf. Sci.* **140**, L239-L243 (1984).

5 Schimka, L. *et al.* Accurate surface and adsorption energies from many-body perturbation theory. *Nat. Mater.* **9**, 741-744 (2010).

6 Paier, J. *et al.* Assessment of correlation energies based on the random-phase approximation. *New J. Phys.* **14**, 043002 (2012).

7 Hammer, B., Hansen, L. B. & Nørskov, J. K. Improved adsorption energetics within density-functional theory using revised Perdew-Burke-Ernzerhof functionals. *Phys. Rev. B* **59**, 7413-7421 (1999).

8 Klimeš, J. & Michaelides, A. Perspective: Advances and challenges in treating van der Waals dispersion forces in density functional theory. *J. Chem. Phys.* **137**, 120901 (2012).
