# Supplementary material for: How Rh surface breaks CO2 molecules under ambient pressure
Source: Nat Commun. 2020 Nov 6;11:5649. doi: 10.1038/s41467-020-19398-1 (PMC7648795; doi:10.1038/s41467-020-19398-1)
Supplement: Supplementary file 3 — Description of Additional Supplementary Files [file 41467_2020_19398_MOESM3_ESM.pdf]

Description of Additional Supplementary Files

File Name: Supplementary Movie 1
Description: Supplementary Movie showing the side view of the normal mode corresponding to the imaginary frequency of the transition state.

File Name: Supplementary Movie 2
Description: Supplementary Movie showing the top view of the normal mode corresponding to the imaginary frequency of the transition state.
